# An empirical study on the intentions and behaviors of non-art majors in continuously selecting esthetic education courses: integration of ECM and TPB

**DOI:** 10.3389/fpsyg.2026.1757284

**Published:** 2026-02-20

**Authors:** Haoran Li, Jiahao Li, Xingchen Fan

**Affiliations:** 1 School of Arts, Sun Yat-sen University, Guangzhou, China; 2 School of Art and Design, Yantai Nanshan University, Yantai, China; 3 Beijing Institute of Technology, Beijing, China

**Keywords:** esthetic education courses, comprehensive universities, continued intention to course selection, expectation-confirmation model, non-art majors, theory of planned behavior

## Abstract

To explore non-art majors’ intentions and behaviors of non-art majors concerning their ongoing enrollment in esthetic education courses, we constructed a theoretical framework integrating ECM and TPB for empirical analysis. The study surveyed undergraduate, master’s, and doctoral students from the comprehensive universities in China. Questionnaires were collected from 519 students selected in esthetic education courses during the second semester of 2024, and integrated structural equation modeling was employed for testing. Analysis reveals that students’ positive attitudes toward esthetic education courses and their perceived feasibility of selection are core variables affecting persistent selection intents. Furthermore, expectation confirmation and satisfaction with esthetic education courses indirectly influence sustained selection intentions. Overall, this study provides evidence that the integrated model offers greater explanatory power for non-art majors’ intentions and behaviors regarding continued selection in esthetic education courses compared to any single model considered in isolation. This establishes a comprehensive theoretical framework for in-depth research on the intentions and behaviors of non-art majors regarding continued selection in esthetic education courses. Simultaneously, it offers practical insights for those aiming to enhance the development, promotion, and management of esthetic education courses at comprehensive universities.

## 1 Introduction

In today’s higher education landscape, where comprehensive student development is increasingly emphasized, esthetic education stands as a vital component of the “five-fold education” framework encompassing moral, intellectual, physical, esthetic, and labor education. Its educational value and functions are becoming increasingly prominent. It not only concerns the cultivation of students’ esthetic abilities but also carries the profound mission of shaping well-rounded personalities, enhancing humanistic literacy, and promoting holistic individual development.

Recently, China has placed high importance on esthetic education. In 2015, the General Office of the State Council issued the “Opinions on Comprehensively Strengthening and Improving Esthetic Education in Schools,” explicitly stating that esthetic education is not only about cultivating esthetic appreciation but also nurturing moral character and spiritual refinement. It elevates one’s esthetic literacy and subtly influences emotions, tastes, temperament, and breadth of mind, inspiring the spirit and enriching the soul (Zhao, 2017). In 2020, the General Office of the CPC Central Committee and the General Office of the State Council jointly issued the “Opinions on Comprehensively Strengthening and Improving School Esthetic Education in the New Era.” This document stressed that school esthetic education is an important way to promote virtue and develop talent. It calls for comprehensively deepening the integrated reform of school esthetic education and establishing a comprehensive esthetic education mechanism for all students (Guo, 2021). Academic explorations have also laid a crucial foundation for understanding the profound value of esthetic education. Du and Li (2024) emphasized that esthetic education in higher education institutions during the new era should focus on the essence of humanistic education, shaping students’ sound personalities and promoting their comprehensive development. They also said that esthetic education is a “comprehensive intermediary” that connects and coordinates with other areas of education (Du and Li, 2024). Chen (2024) similarly noted that esthetic education courses are an important pathway for enhancing students’ esthetic and humanistic literacy (Chen, 2024).

For non-art majors attending comprehensive universities, esthetic education courses hold particular significance. These students typically lack systematic artistic training backgrounds. Such courses provide invaluable opportunities to engage with art, enhance esthetic appreciation, and cultivate humanistic literacy (Fan et al., 2024). Generally, esthetic education courses primarily target comprehensive universities, serving as integrated curricula centered on art education. They combine theory with practice to elevate students’ artistic literacy, esthetic abilities, and creativity. Currently, academic understanding of esthetic education is gradually maturing. Pan (2008) argue that esthetic education should encompass two fundamental dimensions: esthetic education and esthetic-oriented education. The former treats students as esthetic subjects, while the latter views them as esthetic objects. They emphasize that the planning, development, construction, implementation, and management of esthetic education courses should follow a comprehensive esthetic education system to achieve holistic educational objectives (Pan, 2008). Chen (2024) further notes that under the new liberal arts framework, higher education esthetic education curricula must promote cross-disciplinary integration through multiple channels, fully leverage the emotional functions of esthetic education, and enhance its collaborative attributes in nurturing students (Chen, 2024).

However, for a long time, esthetic education has often been regarded as a “soft” course within the higher education system. The status of the course, resource allocation, and the evaluation mechanisms for teaching effectiveness still face numerous challenges. Particularly for non-art majors, the selection of esthetic education courses often involves a degree of autonomy and choice. The willingness and behavior of non-art major university students to continue choosing other esthetic education courses in subsequent semesters, based on their own learning experiences, subjective evaluations, and satisfaction after completing an elective esthetic education course, is referred to as a continuous selection of esthetic education courses. In this context, whether non-art major university students can maintain their willingness to continue selecting courses and put it into practice after taking esthetic education courses is an important challenge currently faced by esthetic education in universities. Pang (2005) noted that public art education in universities faces difficulties such as low status, inadequate institutional frameworks, inefficient systems, and a lack of policy support and institutional safeguards. A lack of top-level design for esthetic education courses has resulted in subpar quality, causing artistic activities to deviate from their original goal of “popularization” (Pang, 2005).

These issues may all impact students’ sustained engagement with esthetic education courses. Against this backdrop, understanding the willingness and behavior of non-art majors to continue selecting in esthetic education courses holds significant theoretical and practical implications for optimizing the esthetic education system in higher education, enhancing the effectiveness of such programs, and promoting students’ well-rounded development. Previous research has predominantly focused on the necessity of esthetic education courses, pedagogical reforms, or students’ motivations for initial course selection. However, there has been a lack of in-depth empirical exploration into whether students, after taking one esthetic education course, develop the willingness and behavior to continue selecting in other such courses based on their experiences and satisfaction levels. This “continuous enrollment” behavior serves as a key indicator for measuring the appeal, teaching quality, and educational effectiveness of esthetic education courses.

To gain a deeper understanding of non-art majors’ willingness and behavior in continuously selecting in esthetic education courses, this study proposes to draw upon two significant theoretical frameworks from social psychology—the theory of planned behavior (TPB) and the expectation-confirmation model (ECM)—and attempts to integrate them organically. The TPB proposed by Ajzen, primarily explains and predicts individuals’ behavioral intentions and actions in specific contexts, positing that behavioral intentions are influenced by attitudes, subjective norms, and perceived behavioral control (Duan and Jiang, 2008; Yan, 2014; Ajzen, 2020). This theory demonstrates strong explanatory power in understanding initial behavioral decisions. However, its explanatory capacity may be limited when assessing whether individuals will persist in a behavior after having performed it once. In contrast, the ECM focus on explaining the mechanism by which users form intentions to continue using a product or service after initial exposure. This intention is based on the degree of confirmation of expectations and satisfaction (Zhang et al., 2016; Yan and Yan, 2013).

Given that non-art majors’ sustained enrollment in esthetic education courses constitutes a dynamic process—involving both initial course selection decisions and subsequent re-evaluations following course experiences—integrating TPB and ECM can provide a more comprehensive theoretical framework. This framework encompasses multiple dimensions, from pre-enrollment motivations and perceptions to post-enrollment experiences and satisfaction levels, thereby revealing the underlying mechanisms driving non-art majors’ intentions and behaviors regarding continued enrollment in esthetic education courses.

## 2 Research model and hypotheses

### 2.1 The expectation-confirmation model

This model was initially proposed by Oliver (1980) to explain consumer satisfaction and repeat purchase behavior. Later, Bhattacherjee (2001) introduced it into the field of information systems, developing it into the expectation-confirmation Model (ECM) to interpret users’ willingness to continue using information systems (Bhattacherjee, 2001; Oliver, 1980). Its fundamental logic posits that users form certain expectations regarding the performance and effectiveness of a product or service prior to use. After use, they compare their actual experience with these initial expectations, leading to confirmation The level of expectation confirmation affects how useful and satisfying users think the product or service is, which in turn affects whether they want to keep using it.

Recently, ECM has been widely applied in the field of education, such as in researching students’ willingness to continue using online learning platforms, blended learning, or other educational technologies (Dou et al., 2025; Obeid et al., 2024; She et al., 2024). Expectation describes how a user anticipates the performance, functionality, or effectiveness of a product or service before using it (Bhattacherjee, 2001). In the context of esthetic education courses, non-art majors may expect the course to enhance their esthetic abilities, enrich their extracurricular lives, or provide enjoyable artistic experiences. Course promotions, instructor introductions, or peer reviews can shape these expectations. Confirmation refers to the degree to which users’ actual experiences align with their initial expectations after using a product or service (Bhattacherjee, 2001). If students observe that the esthetic education course meets or exceeds their initial expectations, their level of expectation-confirmation will be higher. Perceived usefulness refers to the extent to which users believe a product or service helps them achieve their goals (Bhattacherjee, 2001).

Students in esthetic education classes might think that the curriculum is helping them become more esthetically literate, opening their minds, or improving their artistic skills. This perception strengthens their perceived usefulness. Satisfaction refers to users’ overall emotional evaluation of a product or service based on actual experience (Bhattacherjee, 2001). If students are satisfied with the teaching quality, content design, and learning experience of esthetic education courses, they are more likely to develop a willingness to continue selecting. Continuance intention refers to a user’s willingness to continue using a product or service after initial exposure (Bhattacherjee, 2001). As the core dependent variable in ECM, it reflects whether users intend to engage in similar behaviors in the future. In this study, continuance intention manifests as non-art majors’ willingness to selectl in additional esthetic education courses after completing one. Zhang et al. (2016) developed an enhanced model based on ECM when examining MOOC continuance learning intentions. They found that the degree of expectation-confirmation positively influenced learning satisfaction and perceived usefulness experiences. Conversely, satisfaction and perceived usefulness further positively impacted continuance learning intentions (Zhang et al., 2016).

Yan and Yan (2013) also validated this logic in their study on the intention to continue using electronic resources in university libraries. They found that expectation-confirmation is a key predictor influencing perceived usefulness and user satisfaction, which in turn affects the intention to continue using (Yan and Yan, 2013). Additionally, Davis proposed that perceived ease of use and perceived enjoyment are also important factors influencing confirmation expectancy. Perceived ease of use refers to the degree to which an individual believes using a specific system will not be effortful (Davis, 1989). This aligns with students’ perception that selecting and completing esthetic education courses requires minimal additional effort. Perceived enjoyment denotes the degree to which the activity itself is considered pleasurable (Davis et al., 1992). Students experience visual enjoyment and esthetic pleasure during esthetic education courses. Based on this, the study proposes the following hypotheses:

*H*1. Confirmation significantly influences satisfaction.

*H*2. Satisfaction significantly influences continued intention to course selection.

*H*3. Confirmation significantly influences perceived usefulness.

*H*4. Confirmation significantly influences perceived ease of use.

*H*5. Confirmation significantly influences perceived enjoyment.

Although ECM excels at explaining sustained usage behavior, it also has certain limitations. First, ECM primarily focuses on post-experience satisfaction and perceived usefulness. This type of analysis may overlook the complexity of initial behavioral decisions, such as factors like social pressure or personal attitudes.

Secondly, ECM primarily concentrates its applicability on continuous usage behaviors related to technology. This may require further adjustment for non-technical behaviors. Wandira et al. (2024), in their study of cloud-based academic information systems, integrated the technology acceptance model (TAM) with ECM. They found that satisfaction, perceived usefulness, perceived ease of use, and enabling conditions all significantly influence continuous usage intention. This demonstrates that combining ECM with other theories can enhance its explanatory power (Wandira et al., 2024).

Furthermore, most ECM research relies on cross-sectional data from a single point, with limited attention paid to the dynamic evolution of behaviors. Studies by Ye et al. (2022) indicate that the effects of expectation confirmation may vary depending on course type or learning context (Ye et al., 2022). This suggests that when examining esthetic education courses, we must consider the diversity of course content and its impact on student expectations.

In summary, ECM offers a dynamic perspective for understanding non-art majors’ willingness and behavior in continuously selecting in esthetic education courses. It emphasizes the role of post-course validation, satisfaction, perceived usefulness, perceived ease of use, and perceived enjoyment in sustaining behavior. However, its limitations lie in insufficient explanation of initial behavioral decisions and the need for further validation regarding its applicability to non-technical behaviors. Therefore, integrating ECM with TPB can compensate for their respective shortcomings, forming a more comprehensive theoretical framework.

### 2.2 Theory of planned behavior

The theory of planned behavior (TPB) is one of the most renowned and widely applied theories in social psychology concerning the relationship between attitudes and behavior. It was developed by Ajzen based on the Theory of Reasoned Action (TRA). Ajzen (2020) further elaborated on the distinctions between TPB and TRA in his work, emphasizing that the introduction of perceived behavioral control (PBC) represents a key extension of TPB relative to TRA. This extension enables TPB to better explain behaviors that are not fully under volitional control (Ajzen, 2020).

TPB has gained widespread application in the field of educational and learning behavior research due to its robust predictive power. It provides a powerful theoretical tool for understanding students’ learning intentions and behaviors across diverse contexts. These applications validate the universality of TPB and offer significant insights for this study in understanding non-art majors’ willingness to continuously selectl in esthetic education courses.

Specifically, TPB comprises three core components: attitude, subjective norms, and perceived behavioral control. Attitude refers to an individual’s positive or negative evaluation of performing a specific behavior (Ajzen, 1991). Research by Davis et al. (1992) and Lee (2010) explains that perceived usefulness, perceived ease of use, and perceived enjoyment significantly influence attitudes toward technology acceptance (Davis et al., 1992; Lee, 2010). When a technology makes users feel they benefit without excessive effort and brings them enjoyment and pleasure, they develop an intrinsic motivation to adopt it.

As mentioned above, esthetic education courses typically require only sensory and emotional engagement, and students often derive significant visual enjoyment and esthetic experiences from them. Therefore, we can anticipate that perceived usefulness, perceived ease of use, and perceived enjoyment will enhance students’ emotional attitudes toward esthetic education courses and increase their willingness to continue selecting in them. Based on this, the study proposes the following hypotheses:

*H*6. Perceived usefulness significantly influences course selection attitude.

*H*7. Perceived ease of use significantly influences course selection attitude.

*H*8. Perceived enjoyment significantly influences course selection attitude.

*H*9. Course selection attitude significantly influences continued intention to course selection.

Subjective norm refers to the perceived social pressure an individual experiences, specifically whether significant others (such as family members, teachers, friends, social groups, etc.) support or oppose their engagement in a particular behavior (Ye et al., 2022). Hsu et al. proposed that subjective norms are formed by interpersonal influences and external sources of influence. Both exert significant effects on sustained usage intentions (Hsu et al., 2004). Interpersonal influence refers to the impact of family, friends, and other relevant individuals within social networks. External source influence pertains to the impact of mass media, such as television, newspapers, and the internet (Brown and Venkatesh, 2005). Regarding students’ enrollment in esthetic education courses, support from family, teachers, or friends constitutes the interpersonal influence component. Course promotion and previous students’ evaluations of instructors and courses shape external source influence. The research model proposes the following hypotheses to account for these two potential external variables:

*H*10. Interpersonal influence significantly affects subjective norms.

*H*11. External source influence significantly affects subjective norms.

*H*12. Subjective norms significantly affect continued intention to course selection.

Perceived behavioral control refers to an individual’s perception of the ease or difficulty of performing a specific behavior and their sense of control over it (Ajzen, 1991). Su et al. (2021) integrated Perceived University Support with TPB to explore factors influencing entrepreneurial intentions among Chinese university students. Their finresearch indicated that perceived university support had a substantial impact on students’ behavioral control regardingtrepreneurship (Su et al., 2021). This result provides important implications for the present study: “university support” in esthetic education courses (e.g., course resources, faculty expertise, campus cultural atmosphere) may also significantly influence non-arts majors’ perceived behavioral control toward esthetic education courses. This, in turn, affects their willingness to continue selecting in such courses. Therefore, the study proposes the following hypothesis:

*H*13. Perceived behavioral control significantly influences continued intention to course selection.

Overall, the application of TPB in educational and learning behavior research has fully demonstrated its effectiveness in predicting individual behavioral intentions. These studies validate the importance of attitude, subjective norm, and perceived behavioral control as core predictive variables. They also showcase TPB’s potential to enhance explanatory power by integrating external variables. TPB offers a strong theoretical foundation for understanding how non-art majors form intentions to persistently selectl in esthetic education courses before or early in the course selection process. Such choices are based on evaluations of esthetic education courses, social influence, and perceived self-control.

However, TPB primarily focuses on the formation of behavioral intentions, with relatively limited explanatory power regarding the persistence of behavior after it occurs, as well as the influence of experiences and satisfaction on future behavior. This is precisely the aspect that ECM can address.

**Figure 1:**
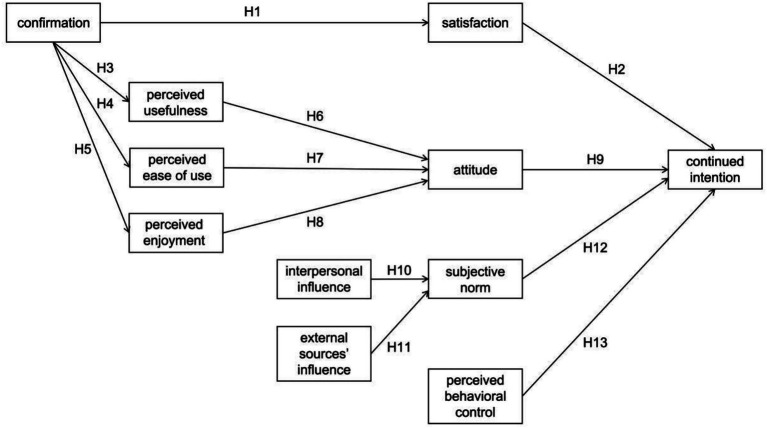
Research model and hypotheses.

Based on the integration of ECM and TPB, the research model and hypotheses are presented in Figure 1.

### 2.3 Integration of ECM and TPB

The integration of ECM and TPB has been validated across multiple domains, particularly in technology adoption and educational behavior studies. For instance, Venkatesh et al. (2011) combined UTAUT predictors (such as effort expectancy, social influence, and facilitators) with ECM when examining sustained use of e-government technologies. They found that expectation-confirmation indirectly influenced intention for sustained use through perceived usefulness and satisfaction. On the other hand, contextual factors (e.g., trust) further enhanced the model’s explanatory power (Venkatesh et al., 2011). Similarly, Wandira et al. integrated ECM and TPB to examine usage intentions toward cloud-based academic information systems, revealing that satisfaction, perceived usefulness, and perceived ease of use collectively drive behavioral intentions (Wandira et al., 2024). These studies demonstrate that integrating TPB and ECM can provide more comprehensive behavioral prediction models by combining psychological factors influencing initial decisions with experiential factors following usage.

Within the context of esthetic education courses, integrating the Extrinsic Motivation Concept (ECM) and Theory of Planned Behavior (TPB) can establish a theoretical framework encompassing initial course selection decisions, course experiences, and sustained enrollment intentions. Specifically, the three core variables of TPB—attitude, subjective norm, and perceived behavioral control—can be used to explain students’ intentions to selectl in esthetic education courses for the first time. Perceptions of the courses’ value, such as enhancing esthetic literacy and enriching extracurricular life, may influence students’ attitudes toward esthetic education courses. Subjective norms may stem from support and expectations of peers, teachers, or parents. Perceived behavioral control may involve course accessibility, scheduling flexibility, and students’ learning capabilities.

## 3 Research methodology

### 3.1 Questionnaire design

We adopted all studies and models from prior research, making minor adjustments to adapt them to the context of elective esthetic education courses. The questionnaire design for this study comprised two main sections. The first part was a demographic survey that asked for basic information about the students, such as their school, gender, grade level, and major. The second part measured latent variables using a 5-point Likert scale. Specifically, the scales for identification, satisfaction, and continuance intention were adapted from Bhattacherjee (2001), while the perceived usefulness scale was modified from Sun (2025). Scales for perceived ease of use, perceived enjoyment, and attitude were adapted from Davis (1989). The social norm scale was adapted from Mathieson (1991). The scales for interpersonal influence and external source influence were derived from Brown and Venkatesh (2005). The scale for perceived behavioral control was adapted from Taylor and Todd (1995a, 1995b). All latent variable scales in this study refer to internationally established scales, with appropriate modifications made for the specific context and research practices.

### 3.2 Data collection

The survey questionnaire for this study was collected online via the Questionstar platform. This platform provides services comparable to Amazon Mechanical Turk. We selectled participating students in our Esthetic Education course during the second semester of the 2024 academic year. Before the final session of the esthetic education course concluded, students were asked to scan a pre-generated QR code displayed on a large screen to access and complete the questionnaire. The survey remained open from May 5, 2024, until June 5, 2024, spanning 1 month. No specific incentives were offered; instead, the questionnaire stated that valid responses would assist faculty and institutions in enhancing esthetic education programs. Students completed a total of 536 questionnaires. Based on three criteria—completion time, completion rate, and consistency in responses to identical questions—we retained 519 valid questionnaires (Table 1).

**Table 1:** Students information.

Variable	Category	Distribution	Valid percentage (%)
University	Sun Yat-sen University	186	35.8
	Beijing Institute of Technology	170	32.8
	Yantai Nanshan University	163	31.4
Gender	Male	272	52.4
	Female	247	47.6
Year level	First Year Undergraduate	90	17.4
	Second Year Undergraduate	102	19.7
	Third Year Undergraduate	80	15.4
	Fourth Year Undergraduate	2	0.4
	First Year Postgraduate	85	16.4
	Second Year Postgraduate	44	8.5
	Third Year Postgraduate	0	0
	First Year Doctoral Candidate	78	14.9
	Second Year Doctoral Candidate	33	6.4
	Third Year Doctoral Candidate	5	0.9
	Fourth Year Doctoral Candidate	0	0
Professional categories	Humanities	226	43.6
	Science	190	36.6
	Engineering	103	19.8

### 3.3 Data analysis

In this study, SPSS version 24 and AMOS version 23 software were employed for further data analysis. Following the two-step approach recommended by Anderson and Gerbing (1988), a confirmatory factor analysis was conducted using maximum likelihood estimation to generate a measurement model for assessing measurement validity. Subsequently, a structural model was constructed to test the proposed theoretical framework.

## 4 Results

### 4.1 Reliability and validity tests

This study first examined the reliability and validity of the scale, with validity subdivided into convergent validity and discriminant validity.

#### 4.1.1 Reliability test

Reliability refers to the degree to which the measurement of a variable is dependable. It is typically assessed using Cronbach’s alpha (CA) and composite reliability (CR). As shown in Table 1, the CA values for each variable in this study ranged from 0.863 to 0.900, while CR values ranged from 0.871 to 0.901, all exceeding 0.700. Therefore, all variables demonstrate satisfactory reliability with their respective measurement indicators.

#### 4.1.2 Convergent validity test

Convergent validity indicates the degree to which theoretically related measurement indicators in a model are actually correlated. That is, it measures the correlation among measurement indicators of a specific variable, primarily assessed using factor loadings (FL) and average variance extracted (AVE), with results shown in Table 2. The factor loadings for all measurement indicators in this study’s questionnaire ranged from 0.759 to 0.963, exceeding 0.700. The average variance extraction values were all above 0.5. Therefore, each variable exhibits excellent convergent validity with its corresponding measurement indicators.

**Table 2:** Reliability and convergent validity.

Variable	Items	FL>0.7	CA>0.7	CR>0.7	AVE>0.5
Perceived usefulness	PU1	0.963	0.900	0.901	0.698
	PU2	0.803			
	PU3	0.788			
	PU4	0.772			
Perceived ease of use	PEOU1	0.936	0.869	0.876	0.703
	PEOU2	0.789			
	PEOU3	0.780			
Perceived enjoyment	PE1	0.927	0.875	0.879	0.709
	PE2	0.795			
	PE3	0.798			
Confirmation	CON1	0.949	0.886	0.887	0.725
	CON2	0.801			
	CON3	0.795			
Attitude	ATT1	0.953	0.881	0.886	0.722
	ATT2	0.793			
	ATT3	0.793			
Subjective norm	SN1	0.921	0.869	0.873	0.697
	SN2	0.783			
	SN3	0.794			
Interpersonal influence	II1	0.925	0.867	0.872	0.696
	II2	0.791			
	II3	0.780			
External sources’ influence	ESI1	0.946	0.883	0.887	0.724
	ESI2	0.800			
	ESI3	0.799			
Perceived behavior control	PBC1	0.952	0.863	0.871	0.695
	PBC2	0.776			
	PBC3	0.759			
Continued intention	CI1	0.939	0.879	0.884	0.718
	CI2	0.797			
	CI3	0.798			
Satisfaction	SAT1	0.957	0.894	0.898	0.748
	SAT2	0.825			
	SAT3	0.804			

#### 4.1.3 Test of discriminant validity

Discriminant validity indicates the degree of differentiation between a specific variable and other variables. It is primarily assessed by comparing the square root of the average variance explained (AVE) with the correlation coefficients, as shown in Table 3. The square root of the AVE for each variable is greater than the correlation coefficient between that variable and other variables. This result demonstrates that the scale possesses discriminant validity.

**Table 3:** Discriminant validity.

	Mean	SD	PU	PEOU	PE	CON	ATT	SN	II	ESI	PBC	CI	SAT
PU	3.549	1.097	**0.835**										
PEOU	3.324	1.069	0.475	**0.838**									
PE	3.392	1.027	0.573	0.427	**0.842**								
CON	3.225	1.126	0.554	0.407	0.437	**0.851**							
ATT	3.696	1.145	0.599	0.412	0.488	0.481	**0.850**						
SN	3.258	1.041	0.558	0.481	0.453	0.404	0.491	**0.835**					
II	3.416	1.011	0.569	0.458	0.397	0.442	0.48	0.497	**0.834**				
ESI	3.306	1.087	0.539	0.440	0.518	0.458	0.509	0.510	0.465	**0.851**			
PBC	3.733	1.085	0.588	0.424	0.410	0.511	0.421	0.449	0.411	0.414	**0.834**		
CI	3.606	1.181	0.603	0.459	0.433	0.387	0.505	0.502	0.461	0.452	0.496	**0.847**	
SAT	3.443	1.177	0.561	0.419	0.450	0.541	0.499	0.482	0.406	0.489	0.466	0.490	**0.865**

The bolded values on the diagonal represent the square roots of the AVE for each construct, while the off-diagonal values below the diagonal indicate the correlation coefficients between the constructs.

### 4.2 Common method bias test

This study employed a questionnaire survey method for data collection. The homogeneity of data from this collection method can lead to common method bias. Therefore, the Harman single-factor test was used to examine whether common method bias existed in the measurement. The specific procedure was as follows: All measurement items were loaded onto a single common factor to construct a single-factor structural equation model. Confirmatory factor analysis was conducted using Amos 23.0. Results indicated poor overall model fit, with the following fit indices: χ^2^/df = 11.292 > 5; CFI = 0.554 < 0.850; NFI = 0.532 < 0.850; TLI = 0.525 < 0.850; IFI = 0.555 < 0.850; RMSEA = 0.141 > 0.08. These findings indicate no significant common method bias.

### 4.3 Model fit indicators

Table 4 presents the model fit results for this study. The findings indicate that χ^2^/df = 1.115 < 3; CFI = 0.996 > 0.9; NFI = 0.959 > 0.9; TLI = 0.995 > 0.9; IFI = 0.996 > 0.9; RMSEA = 0.015 < 0.05. These findings indicate that the measurement model exhibits a favorable fit.

**Table 4:** Model fit indices.

Fit index	Measurement model	Structural model	Recommended criteria	Acceptable criteria
χ^2^/df	1.115	2.134	<3	<5
CFI	0.996	0.953	>0.9	>0.85
NFI	0.959	0.915	>0.9	>0.85
TLI	0.995	0.948	>0.9	>0.85
IFI	0.996	0.953	>0.9	>0.85
RMSEA	0.015	0.047	<0.05	<0.08

### 4.4 Structural modeling test

To validate the hypotheses, this study first analyzed the fit indices of the structural equation model. As shown in Table 4, χ^2^/df = 2.134 < 3; CFI = 0.953 > 0.9; NFI = 0.915 > 0.9; TLI = 0.948 > 0.9; IFI = 0.953 > 0.9; RMSEA = 0.047 < 0.05. All six fit indices of the structural model meet the commonly accepted standards for structural equation modeling. This indicates that the constructed structural equation model exhibits a satisfactory fit. Subsequently, the structural equation model was tested using Amos 23.0. The standardized path coefficients and their significance levels are presented in Table 5.

**Table 5:** Structural model results and hypotheses examination.

Hypothesis	Standardized coefficients	C. R.	*p* value	Support or not
H1: confirmation→satisfaction(+)	0.642	11.484	0.000***	Yes
H2: satisfaction→continued intention(+)	0.182	3.621	0.000***	Yes
H3: confirmation→perceived usefulness(+)	0.698	11.853	0.000***	Yes
H4: confirmation→perceived ease of use(+)	0.521	9.319	0.000***	Yes
H5: confirmation→perceived enjoyment(+)	0.568	10.032	0.000***	Yes
H6: perceived usefulness→attitude(+)	0.446	8.282	0.000***	Yes
H7: perceived ease of use→attitude(+)	0.139	3.021	0.003**	Yes
H8: perceived enjoyment→attitude(+)	0.203	4.086	0.000***	Yes
H9: attitude→continued intention(+)	0.238	4.784	0.000***	Yes
H10: interpersonal influence→subjective norm(+)	0.345	6.766	0.000***	Yes
H11: external sources’ influence→subjective norm(+)	0.360	7.192	0.000***	Yes
H12: subjective norm→continued intention(+)	0.228	4.467	0.000***	Yes
H13: perceived behavior control→continued intention(+)	0.240	4.863	0.000***	Yes

**p < 0.01, ***p < 0.001.

Confirmation has a significant positive effect on satisfaction (*β* = 0.642, *p* < 0.001). Satisfaction has a significant positive effect on continued intention (*β* = 0.182, *p* < 0.001). Confirmation has a significant positive effect on perceived usefulness (*β* = 0.698, *p* < 0.001). Confirmation has a significant positive effect on perceived ease of use (*β* = 0.521, *p* < 0.001). Confirmation has a significant positive effect on perceived enjoyment (*β* = 0.568, *p* < 0.001). Perceived usefulness has a significant positive effect on attitude (*β* = 0.446, *p* < 0.001). Perceived ease of use has a significant positive effect on attitude (*β* = 0.139, *p* < 0.01). Perceived enjoyment significantly and positively influenced attitude (*β* = 0.203, *p* < 0.001). Attitude significantly and positively influenced continued intention (*β* = 0.238, *p* < 0.001). Interpersonal influence significantly and positively influenced subjective norm (*β* = 0.345, *p* < 0.001). External sources’ influence significantly and positively affects subjective norm (*β* = 0.360, *p* < 0.001). Subjective norm significantly and positively affects continued intention (*β* = 0.228, *p* < 0.001). Perceived behavior control significantly and positively affects continued intention (*β* = 0.240, *p* < 0.001).

**Figure 2:**
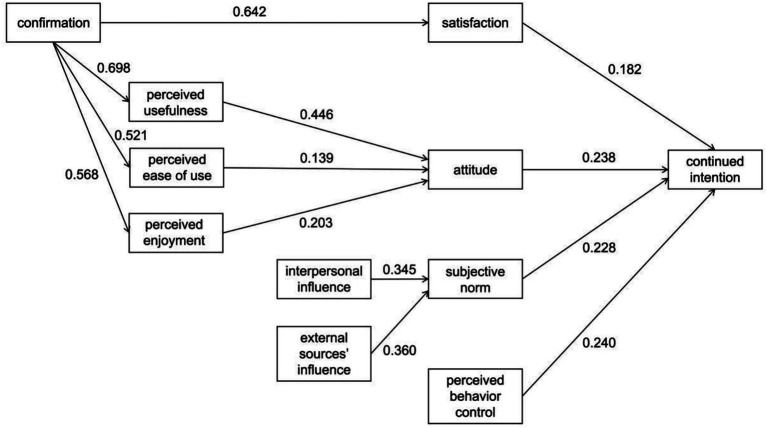
Structural model evaluation.

This indicates that all hypotheses are supported. The R^2^ for satisfaction is 41.3%, for attitude is 39.3%, for subjective norm is 36.5%, and for continued intention is 37.1%, demonstrating that the research model possesses a high degree of explanatory power. Figure 2 presents the measurement results of the overall structural model.

## 5 Discussion and conclusion

This study empirically examines the intention and behavioral mechanisms underlying non-art majors’ sustained enrollment in esthetic education courses, based on ECM integrated with TPB. This section will engage in an in-depth discussion of the findings, elucidating their theoretical contributions and practical implications, before concluding with an acknowledgment of the study’s limitations and suggestions for future research directions.

### 5.1 Discussion

This study confirms the relevance and robust explanatory capacity of the ECM-TPB integrated model in elucidating the enduring learning behavior of non-art majors in esthetic education courses. It also reveals the psychological mechanisms and behavioral logic underlying non-art majors’ continued enrollment in esthetic education courses.

#### 5.1.1 The significant impact of the three key elements of TPB

We examined how attitude, subjective norm, and perceived behavioral control influence the intentions and behaviors of non-art majors regarding their persistent enrollment in esthetic education courses. Findings indicate that perceived behavioral control is the strongest predictor of persistence intention among non-art majors, followed by attitudes and subjective norms as significant yet relatively weaker predictors. These significant effects have previously been validated in studies examining sustained usage behaviors across diverse e-learning contexts (Al-Emran et al., 2020; Li et al., 2022; Shang et al., 2024). Their revalidation within the university setting of continuing enrollment in esthetic education courses further demonstrates the robustness of these associations.

Perceived behavioral control exerted the strongest positive influence on continued intention (*β* = 0.240, *p* < 0.001). This indicates that non-art majors’ decisions to continue selecting in esthetic education courses rely more heavily on their individual assessments of how much control they have over these courses. When they perceive the content difficulty, time conflicts, and resource accessibility of esthetic education courses to be within manageable limits, they demonstrate a significantly stronger intention to continue enrollment.

Attitude exerted a secondary influence on continued intention (*β* = 0.238, *p* < 0.001), indicating that students’ positive evaluations of esthetic education courses serve as an intrinsic motivator for continued enrollment. When they perceive such courses as engaging and capable of enhancing esthetic literacy, they are more likely to continue taking them. The influence of subjective norms on continued enrollment intention was relatively weaker (*β* = 0.228, *p* < 0.001). This indicates that non-art majors’ decisions to continue selecting in esthetic education courses rely more on personal interest than social pressure (e.g., peer recommendations, instructor requirements). It also demonstrates students’ intrinsic motivation for autonomous development in enhancing artistic literacy and esthetic abilities.

#### 5.1.2 Re-verification of the ECM core path

Research findings indicate that confirmation indirectly influences continued course enrollment intention through satisfaction, aligning closely with the conclusions of the classic ECM model. This aligns with empirical studies on users’ continued intention and behavior toward online learning, as well as the persistence of mobile data services. Regarding the correlation between expectation confirmation and satisfaction, Lee’s study reported a coefficient of 0.283 (Lee, 2010). Sumi’s study reported a correlation coefficient of 0.141 (Sumi, 2024). Kim’s study yielded a coefficient of 0.521 (Kim, 2010). Our research demonstrates an even higher coefficient of 0.642. This significantly highlights the strong influence of expected confirmation on satisfaction within esthetic education courses. This indicates that the content and quality of esthetic education courses form the cornerstone for sustaining students’ positive cognitive and emotional engagement. When non-art majors experience esthetic education courses that exceed or meet their initial expectations—such as engaging course content, relaxed teaching styles, pleasant classroom atmospheres, and a strong sense of fulfillment—they exhibit intense positive emotional responses, namely satisfaction. This satisfaction significantly increases their intention and behavior to continue selecting in esthetic education courses.

In this study, we expanded the traditional EMC model by adding two variables to perceived usefulness: perceived ease of use and perceived enjoyment. Confirmation influences attitude by affecting perceived usefulness, perceived ease of use, and perceived enjoyment, which in turn impacts the intention to continue selecting courses.

A more distinct finding is that perceived usefulness emerged as the strongest predictor of continuance intention (*β* = 0.698, *p* < 0.001). This correlation coefficient far exceeded those reported in Lee’s study (0.171) and Sumi’s study (0.014; Lee, 2010; Sumi, 2024). This indicates that when students perceive they can acquire useful new knowledge in esthetic education courses, enhance their esthetic appreciation, and receive academic guidance from instructors, they recognize the course’s value (perceived value). Consequently, they develop a positive attitude toward esthetic education courses, which in turn increases their intention to continue selecting.

The influence of perceived ease of use and perceived enjoyment on attitude was secondary, with correlation coefficients of 0.521 and 0.568, respectively. This indicates that if students experience benefits such as relaxation, visual enjoyment, and stress relief in esthetic education courses, and perceive the content straightforward, they develop positive attitudes and are highly willing to continue selecting. This aligns with Lee’s research findings (Lee, 2010). The correlation coefficient between perceived usefulness and attitude was 0.446, while those for perceived ease of use and perceived enjoyment were 0.139 and 0.203, respectively. This further indicates that students’ strong perception of the course’s usefulness most significantly influences their attitude toward esthetic education courses.

Although the effects of perceived ease of use and perceived enjoyment on attitude were relatively weaker, they reflect the individual perceptions and decision-making characteristics of non-art majors when selecting esthetic education courses.

### 5.2 Conclusion

The study systematically examined the mechanisms underlying non-art majors’ intentions and behaviors in persistently selecting in esthetic education courses by integrating TPB with ECM. Based on the analysis and discussion, the following key findings were identified:

First, among the three components of TPB, the students’ positive attitudes toward serve as key drivers. Core variables influencing sustained selection intentions include students’ positive attitudes toward esthetic education courses (e.g., content appeal, personal interest) and their perceptions of course selection feasibility (e.g., content difficulty, time flexibility, resource accessibility). The role of subjective norms (e.g., peer recommendations, social pressure) is relatively weak. This study indicates that non-art majors primarily base their decision-making on personal interest when selecting esthetic education courses.

Second, the ECM pathway indirectly influences continued course selection intention through expectation confirmation and satisfaction. The degree to which the actual course experience matches expectations (expectation confirmation) indirectly strengthens continued course selection intentions by enhancing satisfaction. However, satisfaction does not have a significant direct effect on continued behavior.

Third, in the integrated model, expectation confirmation indirectly influences attitudes through perceived usefulness, perceived ease of use, and perceived enjoyment. This subsequently affects the intention to continue selecting, demonstrating greater explanatory power: the integrated model combining TPB and ECM sigsignificantly outperforms either model alone in explaining both the intention to continue selecting and actual behavior (R^2^ = 37.1%). This validates the effectiveness of the integrated model.e complementary roles of psychological motivation and experiential feedback within the context of esthetic education courses.

### 5.3 Theoretical contributions and practical implications

#### 5.3.1 Theoretical contributions

The theoretical basis for integrating TPB into ECM lies in the following: The constructs of TPB may primarily influence individuals’ intention formation when they first selection in an esthetic education course. However, after experiencing the course, students’ decisions to continue selecting in subsequent sessions are influenced by feedback from their course experience, which relates to the constructs of ECM.

This feedback mechanism of expectation confirmation and satisfaction will in turn influence students’ attitudes toward esthetic education courses and their perceived behavioral control. It may even indirectly affect their perception of subjective norms, thereby reshaping their willingness to continue selecting in such courses. The integration of TPB and ECM offers the advantage of comprehensively covering the entire behavioral decision-making process—from initial intention to actual behavior and ultimately to the formation of sustained behavior.

The Theory of Planned Behavior (TPB) provides the psychological mechanism for initial course selection decisions, while the Experiential Continuation Motivation (ECM) model supplements the ongoing behavioral motivation based on experience. Together, they offer a more comprehensive explanation of the complex psychological processes driving non-art majors to persistently select in esthetic education courses. For instance, students may initially select due to positive attitudes toward esthetic education courses and peer recommendations (TPB). Nonetheless, the readiness to persist in selection arises solely when the course experience aligns with or surpasses expectations, resulting in satisfaction (ECM).

The advantages of this integrated model are fourfold:

First, it gives a better overall picture of behavior. The integrated model provides a more holistic understanding of the behavioral process, covering the entire chain from the initial formation of intention to subsequent sustained behavior. It not only explains why students initially choose esthetic education courses but, more importantly, why they choose to continue selecting in them. This information is crucial for understanding long-term behavioral patterns.

Secondly, it highlights the importance of dynamicity and feedback mechanisms. The “confirmation” construct introduced by ECM emphasizes the comparison between actual experiences and initial expectations. This feedback mechanism is absent in TPB. Through integration, we can observe how TPB constructs such as students’ attitudes and perceived behavioral control undergo dynamic changes after course experiences, influenced by confirmation and satisfaction. For example, if students’ expectations for esthetic education courses are met and they are happy with them, they may have a better attitude toward these courses. Simultaneously, their perceived behavioral control regarding continued enrollment may also strengthen.

Third, the choice to take esthetic education courses, particularly for non-art majors, involves both an initial intention of autonomous selection and ongoing decisions based on post-experience perceptions and evaluations. The integrated model better accommodates this scenario by capturing students’ psychological decision-making processes across different stages. In the initial stage, students may focus more on the course’s “attractiveness” and “necessity” (TPB). In the ongoing phase, they increasingly weigh the course’s “value” and “experience” (ECM).

Fourth, by integrating the strengths of both models, the combined model is expected to provide greater explanatory power and predictive capability concerning non-art majors’ willingness and behavior to continue selecting in esthetic education courses. For instance, satisfaction within the ECM can directly serve as a strong predictor of “attitude” in the TPB. Alternatively, it can function as an additional variable influencing “behavioral intention,” thereby enhancing the model’s predictive efficacy.

#### 5.3.2 Practical implications

This study proposes the following recommendations for the development, promotion, and management of esthetic education courses in higher education institutions. Firstly, we should continuously optimize course content, innovate teaching methods, and strengthen experiential learning and interdisciplinary integration at the curriculum design level. Enhance course appeal (reinforce attitudes) through modules such as art workshops and lifestyle esthetics practices, while lowering participation barriers (improve perceived behavioral control) by adopting flexible assessment methods and open access to resources (e.g., shared art venues). Second, at the teaching management level, campus esthetic education activities like art events, exhibitions, and lectures should convey the non-utilitarian value of the curriculum (e.g., esthetic literacy, creativity cultivation). Simultaneously, leverage the driving role of student clubs and key opinion leaders (KOLs), to establish a dynamic feedback mechanism. Ultimately, this enables real-time optimization content to enhance expectation confirmation and satisfaction.

### 5.4 Research limitations and future prospects

However, the integrated ECM and TPB model also faces some challenges, and this study has the following limitations, which may affect the generalizability of its conclusions.

First, although the complexity of the model allows for detailed explanations, it also increases the difficulty of data collection and analysis. Future research needs to develop more comprehensive scales to measure the relationships among various variables. Of particular note is that the diversity of esthetic education courses may cause differences in expectation confirmation and satisfaction. Future studies should consider course types as important moderating or control variables.

Second, the cross-sectional design of this study is a key limitation. This design only collects data at a single point, which can reveal correlations between variables but makes it difficult to establish causal relationships and does not allow for dynamic tracking of the evolution from “satisfaction” to “continuance intention.” For example, the impact of initial course experience satisfaction on long-term intention may change over time or with subsequent experiences. Future longitudinal studies will be better able to test the dynamic predictive power of the model.

Third, the study sample was drawn from only three universities, and the characteristics of their regions, school types, and student populations may limit the generalizability of the results. Moreover, this study is rooted in the specific higher education and cultural context of China. Chinese society’s perception of esthetic education, university course offerings and evaluation systems, and the potentially utilitarian learning atmosphere faced by students all create a unique cultural context. These factors may make the mechanisms of variables such as “subjective norms” and “perceived usefulness” culturally specific. Therefore, we must cautiously validate the conclusions of this study when applying them to other cultural or educational contexts.

Finally, to deepen the understanding of these mechanisms, future research could introduce additional variables, such as students’ baseline interest in art, deep learning motivation, or flow experiences, to explore their mediating or moderating roles within the model.

## Data Availability

The original contributions presented in the study are included in the article/Supplementary material, further inquiries can be directed to the corresponding author.
