# Supplementary material for: An empirical study on the intentions and behaviors of non-art majors in continuously selecting esthetic education courses: integration of ECM and TPB
Source: Front Psychol. 2026 Feb 20;17:1757284. doi: 10.3389/fpsyg.2026.1757284 (PMC12963004; doi:10.3389/fpsyg.2026.1757284)
Supplement: Supplementary file 1 [file Supplementary_file_1.docx]

**Appendix A**

**Survey on the Continuous Elective Intention and Behavior of Aesthetic Education Courses Among Non-Art Major College Students**

Dear Students,

Hello! This questionnaire aims to investigate the elective intentions and behaviors of non-art major college students regarding aesthetic education courses. All data will be used solely for academic research and will be kept strictly confidential. There are no right or wrong answers, so please respond based on your true feelings and actual situation. Completing the questionnaire takes about 2-5 minutes. We sincerely appreciate your support and cooperation!

Instructions:

All items in this questionnaire use a 7-point Likert scale, where 1 means strongly disagree, 2 means somewhat disagree, 3 means slightly disagree, 4 means neutral, 5 means slightly agree, 6 means mostly agree, and 7 means strongly agree. Please mark "√" on the corresponding number.

| Questionnaire Body |
| --- |
| \| **Item Dimension** \| **Serial Number** \| **Item Content** \| **1 Strongly Disagree** \| **2 Disagree** \| **3 Somewhat Disagree** \| **4 Neutral** \| **5 Somewhat Agree** \| **6 Agree** \| **7 Strongly Agree** \| \| --- \| --- \| --- \| --- \| --- \| --- \| --- \| --- \| --- \| --- \| \| **Perceived Usefulness** \| 1 \| The content of the aesthetic education courses interests me a lot. \|  \|  \|  \|  \|  \|  \|  \| \|  \| 2 \| I learned new things from the aesthetic education courses. \|  \|  \|  \|  \|  \|  \|  \| \|  \| 3 \| The aesthetic education courses helps improve my aesthetic values. \|  \|  \|  \|  \|  \|  \|  \| \|  \| 4 \| I received academic guidance and aesthetic cultivation from the lecturer. \|  \|  \|  \|  \|  \|  \|  \| \| **Perceived Ease of Use** \| 5 \| I find taking aesthetic education courses very easy. \|  \|  \|  \|  \|  \|  \|  \| \|  \| 6 \| I can easily and skillfully grasp the content of aesthetic education courses. \|  \|  \|  \|  \|  \|  \|  \| \|  \| 7 \| Overall, aesthetic education courses are easy to attend. \|  \|  \|  \|  \|  \|  \|  \| \| **Perceived Enjoyment** \| 8 \| Attending aesthetic education courses is enjoyable. \|  \|  \|  \|  \|  \|  \|  \| \|  \| 9 \| Attending aesthetic education courses is pleasant. \|  \|  \|  \|  \|  \|  \|  \| \|  \| 10 \| I find attending aesthetic education courses very interesting. \|  \|  \|  \|  \|  \|  \|  \| \| **Confirmation** \| 11 \| My experience in the aesthetic education courses was better than I expected. \|  \|  \|  \|  \|  \|  \|  \| \|  \| 12 \| The level of the aesthetic education courses was better than I expected. \|  \|  \|  \|  \|  \|  \|  \| \|  \| 13 \| Overall, most of my expectations for the aesthetic education courses were met. \|  \|  \|  \|  \|  \|  \|  \| \| **Attitude** \| 14 \| Taking an elective aesthetic education courses is really a good idea. \|  \|  \|  \|  \|  \|  \|  \| \|  \| 15 \| I enjoy attending aesthetic education courses. \|  \|  \|  \|  \|  \|  \|  \| \|  \| 16 \| I suggest everyone take an elective aesthetic education courses. \|  \|  \|  \|  \|  \|  \|  \| \| **Subjective Norm** \| 17 \| People important to me hope that I take aesthetic education courses. \|  \|  \|  \|  \|  \|  \|  \| \|  \| 18 \| People who influence my behavior think I should take aesthetic education courses. \|  \|  \|  \|  \|  \|  \|  \| \|  \| 19 \| People whose opinions I value more hope that I take aesthetic education courses. \|  \|  \|  \|  \|  \|  \|  \| \| **Interpersonal Influence** \| 20 \| My family thinks I should take aesthetic education courses. \|  \|  \|  \|  \|  \|  \|  \| \|  \| 21 \| My friends think I should take aesthetic education courses. \|  \|  \|  \|  \|  \|  \|  \| \|  \| 22 \| People I know think taking aesthetic education courses are good idea. \|  \|  \|  \|  \|  \|  \|  \| \| **External source influence** \| 23 \| Information from mass media suggests I take aesthetic education courses. \|  \|  \|  \|  \|  \|  \|  \| \|  \| 24 \| Based on information pushed through mass media, I think I should take aesthetic education courses. \|  \|  \|  \|  \|  \|  \|  \| \|  \| 25 \| According to what I heard or saw in the mass media, I am encouraged to take aesthetic education courses. \|  \|  \|  \|  \|  \|  \|  \| \| **Perceived Behavioral Control** \| 26 \| I can independently choose whether to take aesthetic education courses. \|  \|  \|  \|  \|  \|  \|  \| \|  \| 27 \| The content of aesthetic education courses is completely within my understanding and control. \|  \|  \|  \|  \|  \|  \|  \| \|  \| 28 \| I have sufficient resources, knowledge, and ability to do well in aesthetic education courses. \|  \|  \|  \|  \|  \|  \|  \| \| **Satisfaction** \| 29 \| I am satisfied with my overall experience in aesthetic education courses. \|  \|  \|  \|  \|  \|  \|  \| \|  \| 30 \| I feel very happy during the process of taking aesthetic education courses. \|  \|  \|  \|  \|  \|  \|  \| \|  \| 31 \| I gain aesthetic enjoyment from studying aesthetic education courses. \|  \|  \|  \|  \|  \|  \|  \| \| **continued intention** \| 32 \| I plan to continue taking elective courses related to aesthetic education in the future. \|  \|  \|  \|  \|  \|  \|  \| \|  \| 33 \| I intend to increase my learning efforts in aesthetic education courses going forward. \|  \|  \|  \|  \|  \|  \|  \| \|  \| 34 \| I will maintain the current frequency of taking aesthetic education courses in my subsequent studies. \|  \|  \|  \|  \|  \|  \|  \| |

Thank you once again for taking the time to complete this questionnaire!

Questionnaire website：https://www.wjx.cn/vm/PnqezNj.aspx
